# Size Does Matter: Staging of *Silene latifolia* Floral Buds for Transcriptome Studies

**DOI:** 10.3390/ijms160922027

**Published:** 2015-09-11

**Authors:** Su San Toh, Michael H. Perlin

**Affiliations:** Department of Biology, Program on Disease Resistance, University of Louisville, Louisville, KY 40292, USA

**Keywords:** *Silene latifolia*, *Microbotryum*, anther smut, RNA-Seq, floral bud stage, host-pathogen interactions

## Abstract

Dioecious plants in the Caryophyllaceae family are susceptible to infection by members of the anthericolous smut fungi. In our studies of the *Silene latifolia/Microbotryum lychnidis-dioicae* pathosystem, we were interested in characterizing the plant-pathogen interaction at the molecular level before and during teliosporogenesis. This takes place during floral bud development, and we hoped to capture the interaction by Illumina Next-Gen RNA-Sequencing. Using previous literature that documented the stages of the floral buds for *S. latifolia*, we examined the floral buds from plants grown and infected under growth chamber conditions, using the disserting microscope to determine the stage of floral buds based on the morphology. We compiled the information and determined the size of floral buds that correspond to the desired stages of development for tissue collection, for the purpose of RNA-sequencing. This offers a practical approach for researchers who require a large number of floral buds/tissue categorized by stages of development, ascertaining whether infected/uninfected buds are at comparable stages of development and whether this also holds true for male *vs*. female buds. We also document our experience in infecting the plants and some of the unusual morphologies we observed after infection.

## 1. Introduction

*Silene latifolia*, formerly known as *Melandrium album*, is a dioecious flowering plant, a member of the Caryophyllaceae family. It is one of the most studied species among vascular plants that display sexual dimorphism. *S. latifolia* is also a model system for the study of the evolution of plant sex chromosomes. The sex chromosomes are similar in many ways to the older animal sex chromosome system, but these plant sex chromosomes are relatively early in the evolutionary strata, *i.e*., where the Y chromosome is in its early stages of degeneration [1,2,3,4,5]. Hence, it is a useful model in the elucidation of sex chromosome evolution.

In natural populations of *S. latifolia*, the genus is associated with the fungal phytopathogen, *Microbotryum lychnidis-dioicae*, a member of the *Microbotryum violaceum* fungal complex commonly known as anther smuts due to the dark teliospores found in the infected flowers. The fungus is biotrophic and can only complete its sexual life cycle in the host. The healthy host plants display strong sexual dimorphism in vegetative [6] and reproductive traits [7]. When infected, the fungus replaces the pollen in a male plant; in contrast, the gynoecium is suppressed in infected female flowers, and pseudo-anthers develop to house the teliospores, much like in the male plant [8,9,10]. Infection of females may thus produce at least a partial sex reversal [8,9,10]. The infection renders both the male and female plants sterile, in the former, due to the lack of pollen, while, in the latter, the rudimentary ovary that can no longer set seeds.

When pollinators visit both infected and uninfected flowers for the nectar reward [11], the teliospores are transferred along with pollen [12]. Partial infection may take place in the first year of infection, but systemic infection tends to develop in subsequent years. The deposited teliospores germinate and undergo meiosis, leading to a linear tetrad that produces haploid yeast-like sporidia of opposite mating types, *a*_1_ and *a*_2_ [13], analogous to gametes of the fungus. Conjugation between sporidia of opposite mating types is required to initiate the growth of dikaryotic infectious hyphae [14], which “travel” through the intercellular spaces of the apical meristem of infected plants where they reside and subsequently infect the plant systematically. However, the exact location and mechanism of infection is still unclear [15].

Although the Y-chromosome is the sex-determining factor in the host [16], the genes that enable the development of both the gynoecium and androecium are found on the X-chromosome [17,18], which is why all of the floral buds are “bisexual” or hermaphroditic at the earliest stages of development [19]. The fungus appears to co-develop with the floral buds, such that the developmental stage of the flower bud is an accurate indication of the stage of fungal teliosporogenesis [9,10]. Previous expressed sequence tag (EST) library and RNA-Seq transcriptome studies were conducted, for the most part, from healthy plant floral tissue at less than Stage 10 [19] and mainly for the purpose of finding expression differences between the male and the female plants [20,21,22,23]. One study that examined infected flowers was done with the aim of elucidating specific expression differences in male plants using a cDNA subtraction approach using pooled tissue for Stages 8–11 [24]. Preliminary transcriptome studies identified genes differentially expressed in the fungus during late stage infection of male flowers [25]. With the current next-generation RNA-sequencing technology, we can potentially correlate the interaction of gene expression between the fungus and its host at each stage of their co-development. Such a study [26] would be a first in terms of stage-by-stage analysis of early flower development and early stages of teliosporogenesis for the *S. latifolia*/*M. lychnidis-dioicae* pathosystem. The accurate staging of the floral buds is therefore crucial to the effective discovery of the gene expression interaction between stages of development and the comparison between male and female, infected and uninfected plants. Floral development stages [19,27], in relation to bud size, and the corresponding fungal stages in infected floral tissue [9] had previously been documented (but also see [15], for SEM images). However, we recognized that, in addition to light, temperature and other confounding factors that may affect the development of flowers, infection with *M. lychnidis-dioicae* itself may alter the relationship between bud size and floral developmental stage, especially for the very young floral bud. The overall reduction in inflorescence height, flower size and nectar production has previously been reported [28], indicating that infection by the fungus can indeed alter physical development of the host. Moreover, previous studies found that diseased plants produce smaller flowers with smaller reproductive structures than healthy plants [28,29] and more flowers, especially in females. Thus, after achieving stable infection of the plants serving as sources of tissue for RNA-Seq studies, it was necessary to examine the buds explicitly to stage them according to the laboratory conditions used for propagation. This process was meant to facilitate the rapid and accurate collection of tissue, so as to preserve the integrity of the RNA and to have confidence in the resulting RNA-Seq data.

In devising the infection method, we noted that previous studies showed full germination of teliospores on both seedlings and flowers; with the basidia of such germinations producing promycelia upon meiosis. On seedlings, most promycelia were two-celled compared to four-celled promycelia in the flower. In the two-celled basidia, conjugation hyphae formed rather than basidiospores (*i.e*., sporidia), and subsequently, an infectious hypha with a swollen appressorial structure at the tip of the hypha was observed [15], although the penetration appears to be due to enzymatic processes, rather than via turgor pressure. In contrast, infection in the flower resulted in the basidiospores reproducing via budding, probably because of the higher nutrient availability. Therefore, inoculation of seedlings was preferred. It also eliminated the need to grow the plants to maturity before infection could take place.

We explored various delivery methods reported in the literature for laboratory infection. Cells were either pre-mated [28] or provided as unmated mixtures of the two haploid partners of opposite mating type [30]. Two main modes of delivery were also commonly employed: injection (*i.e*., inoculation accompanied by puncture with a needle) [28,30] and inoculation via application of inoculum as a droplet to undamaged tissue [29,31]. Some reports employed teliospores as inoculum [31], but since we did not have infected plants to start with, we did not try this until much later (not reported in the data here). As the original aim of this work was to obtain robustly-infected plants, each infection experiment was an attempt to obtain more robustly-infected plants, rather than a systematic study of what was the most effective mode of infection.

In the work described in this paper, we attempted to document the various bud sizes of both male and female, infected and uninfected floral buds, first size categorized by hand (which will be the method of bud collection for RNA-sequencing samples) and verifying the size and their floral development under the dissecting microscope. The definition of the stages was informed by graphical and text illustration of previously-published studies [9,10,27].

## 2. Results and Discussion

### 2.1. Infection Method

It was difficult to determine the most successful infection method, since there was no one method that worked for every plant in a single trial in more than 20 batches of plants. Each infection experiment was performed with the aim of obtaining more robustly-infected plants. Some infected plants also showed different “degrees” of infection, which made it hard to determine the infection status.

For example, since smut infection can only be observed clearly when a plant flowers, those that remained as rosettes could not be labeled visually as infected until or unless flowering occurred, unless PCR amplification of diagnostic genes for *M. lychnidis-dioicae* [32] was employed. Such diagnostics were not of practical application in these studies, since the purpose was to accurately determine flower bud stages for potential tissue collection. There were also some plants that bore buds that never bloomed, turned brown or withered before they were ready to bloom, which again made it hard to determine the infection status. Moreover, some plants bore a few bolts that were smutted and a few that were not, and some “lost” the smut infection after some time. Some even simultaneously bore many bolts that never flowered. All of these could be some degree of disease presentation, since uninfected plants almost never displayed similar symptoms. Such symptoms had previously been observed and reported anecdotally [30,33]. Various phenotypic changes were also observed with infection by *M. lychnidis-dioicae* where the infection percentage was rather low [34]. In that particular study, only 912 plants (33.6%) were diseased out of the 2717 that were inoculated and flowered (a total of 3808 were inoculated, and 12% died in the inoculation experiment). Therefore, difficulty with achieving infection may not be uncommon; on the other hand, an acceptable infection rate is usually more than 60% [33].

In our infection experiments (about 20 independent experiments performed over more than two years), 20 plants were injected and 111 plants were inoculated via the application of mated mixtures of the fungus without damage to the shoot apical meristem (treatments were not exclusive). Of these, there was some mortality not attributable to infection (*i.e*., not significantly more than uninfected controls). Ten plants remained in rosette for as long as we had them; all of these had been inoculated, half with pre-mated cells, and the other half received a mixture of unmated cells. None of the control plants remained in the rosette stage. The batch of seeds used in this study was from progeny of healthy individuals in an infected population, grown in the greenhouse. Although the original population had smut infection, we cannot determine whether “innate” immunity plays a role in the difficulty we experienced in infecting the plants. The infection rate of this combination of host and fungus was originally found to be about 50% [35]. Strain-specific resistance has also been suggested [36], but we are unclear whether the difficulties were due to host-pathogen interaction [37] or because of imperfections in the artificial infection method. From all of these observations, we found that pre-mated cells were overall more effective as inocula than unmated cells. The total amount of cells used for inocula ranged from 1 × 10^4^–5 × 10^7^ cells, and there was no correlation in the optimal amount to use (taking into consideration that the infective suspension volume was always in excess, it is reasonable that we were unable to determine an optimal cell count for infections).

Although field studies have showed earlier onset of flowering between healthy male and female plants, no obvious difference was seen between diseased and healthy plants [29]. On the other hand, the same study by Shykoff and Kaltz [29] found host sex-specific differences, with significantly higher infection rates among *S. latifolia* females than males. Similarly, differences in infection rate between male and female plants were observed in field populations of *S. dioica* [38]. In our experiments, there were a total of eight female plants and 13 male plants that were infected (showing smutted flowers at some point), but not all were robustly infected (*i.e*., with long-term, systemic infection and an otherwise “healthy” host), a requirement for the eventual RNA-Seq experiments. Including the plants that did not bloom at all, we estimate an infection rate of less than 25%. This includes plants that never flowered, but that might have been infected and presented symptoms not observed in uninoculated plants. Also included in this group were four plants that presented with unusual flower morphologies (see Section 2.4, below). This lower overall infection rate probably reflects the variety of methods we tested before an optimal approach was reached.

### 2.2. Floral Size and Staging

**Figure 1:**
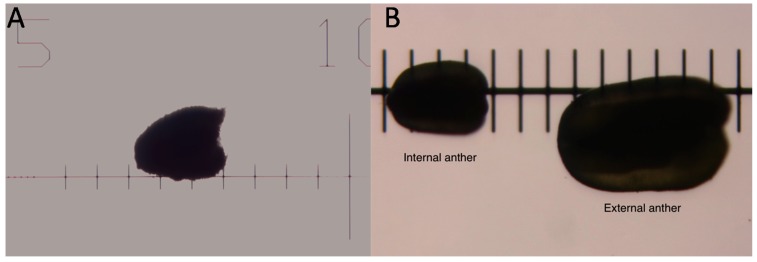
Illustration of how the measurements of floral bud (**A**) and anthers (**B**) were taken using a glass stage micrometer. Each division is 1 mm in (**A**) and 0.1 mm in (**B**). These pictures were taken in the light field, but measurements were typically taken in the dark field.

The purpose of staging the floral buds was to develop a quick way of determining the floral buds that correspond to the desired floral developmental stage to be collected for RNA-sequencing, especially when examining the interaction of the fungus and host. Therefore, only the robustly-infected plants and corresponding uninfected plants were used. Figure 1 illustrates how measurements were taken under the dissecting scope on the glass micrometer. The goal for these studies was, thus, to assure or to determine the correspondence between infected and uninfected bud sizes and stages of floral development. Only plants used as controls in the infection experiments were subsequently taken as tissue sources for the uninfected plants in staging here and later, in the RNA-Seq experiments.

**Figure 2:**
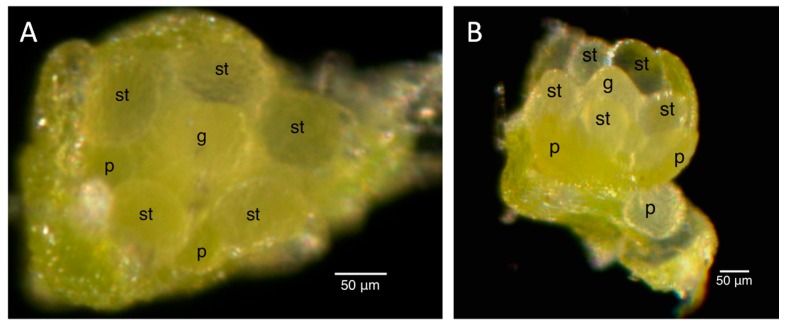
Stage 6 of 0.2-mm (**A**) and 0.25-mm (**B**) male infected floral bud with unilobal anthers and petals that were observed as small molds. “Mushroom”-shaped gynoecium can be observed here. Size bars, approximately 50 µm. g, gynoecium; st, stamen; p, petal.

**Figure 3:**
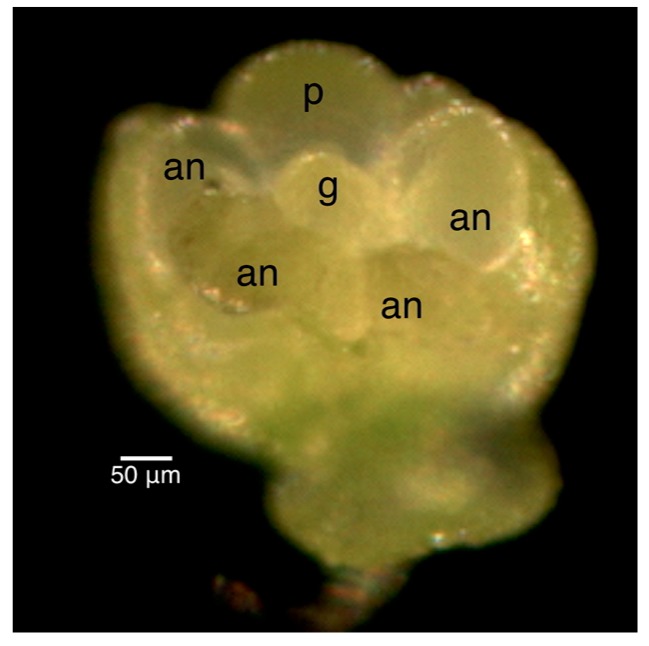
Stage 7 of 0.3 mm male infected floral bud showing bilobal external anthers and petals like small molds. Size bars, approximately 50 µm. g, gynoecium; an, anther; p, petal.

**Figure 4:**
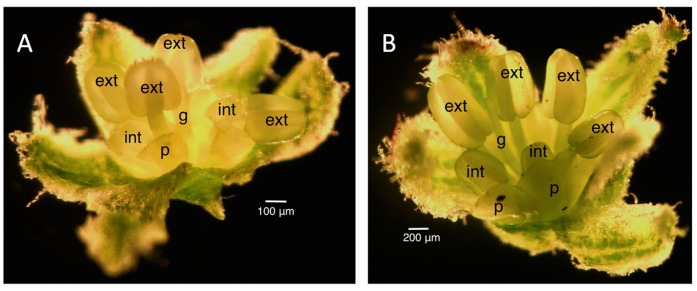
Stages 8 (**A**) and 9 (**B**) male infected floral buds. Stage 9 buds have tetralobal internal anthers and pale green, cordiform petals compared to bilobal internal anthers and translucent, tongue-like petals in Stage 8. The gynoecium is developing into a finger-like projection. Size bars, approximately 100 and 200 µm, respectively, in (**A**,**B**). ext, external anthers; int, internal anthers; g, gynoecium; p, petal.

The staging for male buds depended on the morphology and size of the anthers and the morphology of the petals. Once the stages were established in the male infected floral buds, the male uninfected floral buds were sampled to ensure that the staging would correspond appropriately to the uninfected plant. Morphologically, the stamen first looked like a ball, known as the unilobal anther (Figure 2). The ball then “split” into two parts to form the bilobal anther (Figure 3) and, finally, into four parts, to form the tetralobal anther (Figure 4). The petals were observed as small molds alternating with the sepal at about Stage 7 (Figure 3) and developed into tongue-like structures at Stage 8 (Figure 4A). As it grew larger, a notch formed in the center, yielding a heart-shaped structure or cordiform (Figure 4B). Development of the gynoecium was arrested and became a finger-like projection; thus, the gynoecium was not used as a structure for staging males.

**Figure 5:**
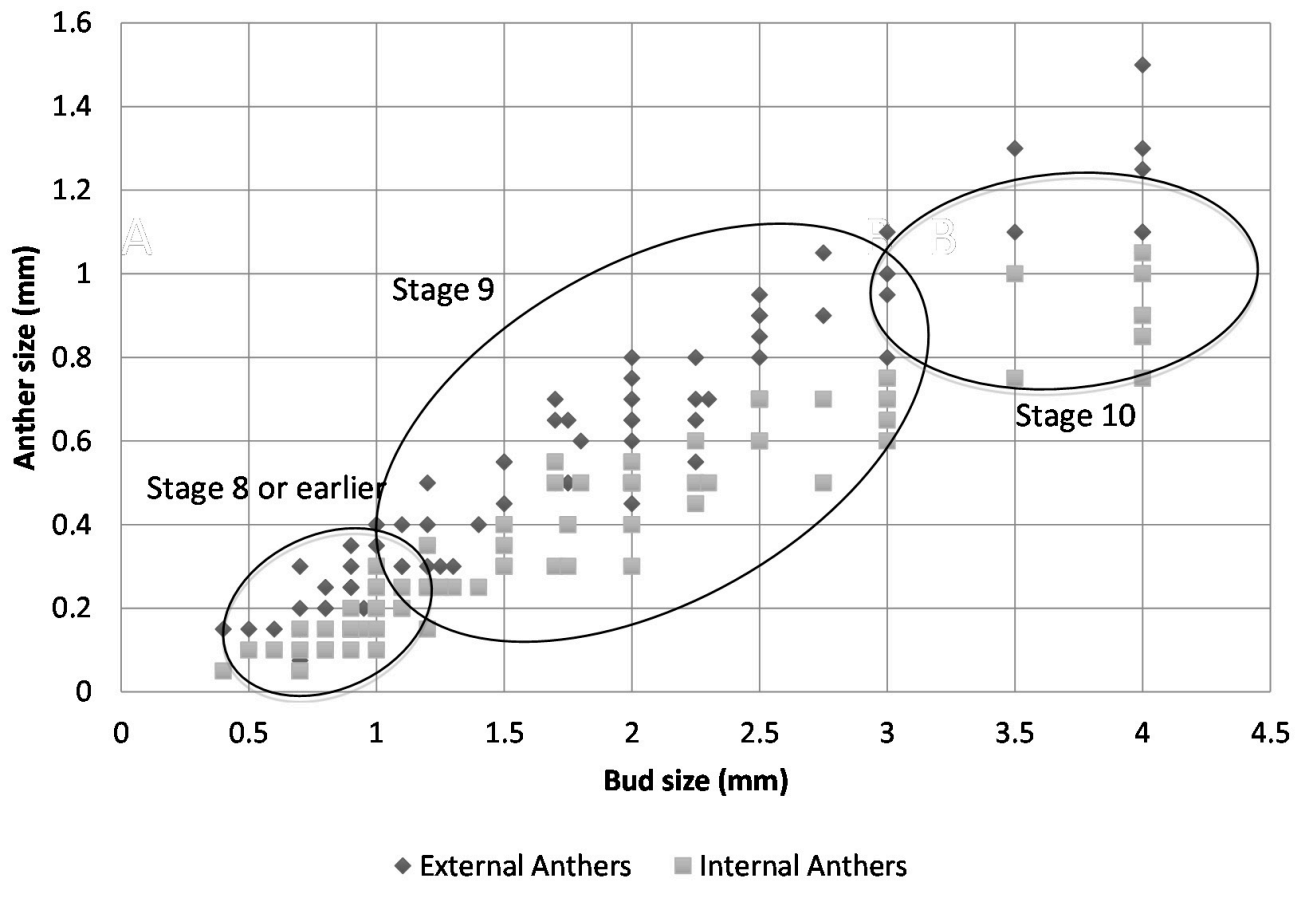
Scatterplot of the external and internal anther sizes of the infected and uninfected floral buds against the size of the floral buds. Ellipsoids indicate the range in bud sizes for flowers of the respective developmental stage groups indicated.

**Figure 6:**
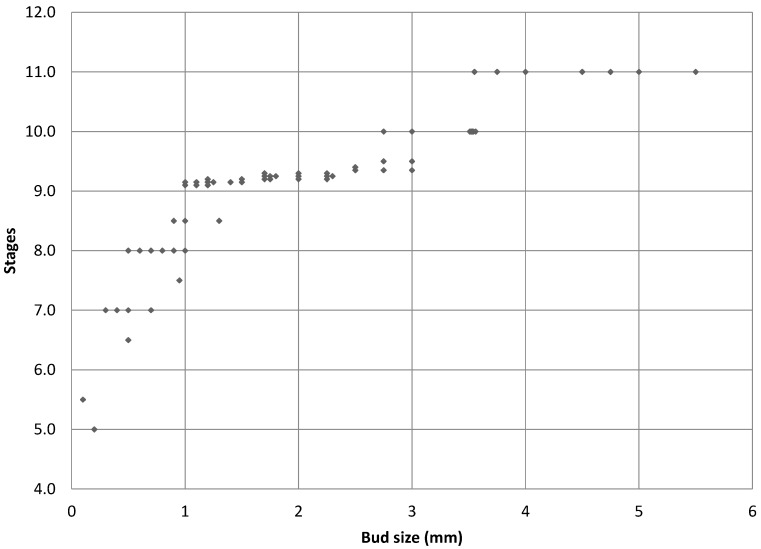
Scatterplot of male infected bud floral stage against the size of the floral buds.

Figure 5 shows a scatter plot of the sizes of the external and internal anthers against the bud size. At Stage 8, external anthers should be 0.2 mm and internal anthers should be 0.1 mm. At Stage 9, the external anthers measure 0.3–1 mm and the internal anthers 0.2–0.7 mm. Stage 10 anthers measure 1.1 mm (external) and 0.8 mm (internal), respectively. Based on the plot, we can estimate the sizes of buds for each stage of interest (the morphology of anthers and petals was also considered in making the determination). Figure 6 presents the data in a way that may further inform this strategy for bud selection, by plotting floral stages against bud sizes. Although there is some overlap of stages, this scatterplot analysis clearly shows separation of Stages 10 and 11 (at a 3.5-mm bud size), whereas Stages 8 and 9 overlap (around 1 mm) as do Stages 9 and 10 (around 3 mm) at some point over the range of bud sizes collected. In summary, for the male plants, the smallest bud that could be isolated from a cluster was 0.3 mm (determined under a dissecting scope), and such buds were usually hidden in the sepal. Therefore, the sepals were collected as the <1-mm group after all buds larger than 1 mm were removed. Based on our measurements, these buds were clearly Stage 8 [9,10,27] or earlier in floral development (Figure 2, Figure 3 and Figure 4A). This is consistent with the reference literature [9,10,27]. Another quick way to determine the cutoff for Stage 8 floral buds during tissue collection was the appearance of trichome on the calyx. Stage 9 (Figure 4B) of floral development was long and was determined in our population to be floral buds of a size larger than 1 mm, but smaller than 3 mm. In the male plants we examined, the maturation of anthers from bilobal to tetralobal was the main change. The petal also started to develop a notch in the tongue-like petal to look like a cordiform, with the color changing to pale green. Stage 9 for these male plants lasted a little longer than in the reference literature, where 2.8-mm buds were already at Stage 10. In our plants, Stage 10 only started around 3 mm and lasted a very short period, until just below 4 mm. The brevity of Stage 10 posed a challenge in tissue collection in having enough Stage 10 floral buds for RNA-Seq analyses. Floral buds larger than 4 mm through bloomed flowers were determined to be Stage 11 and beyond and were broadly categorized as late stage.

**Figure 7:**
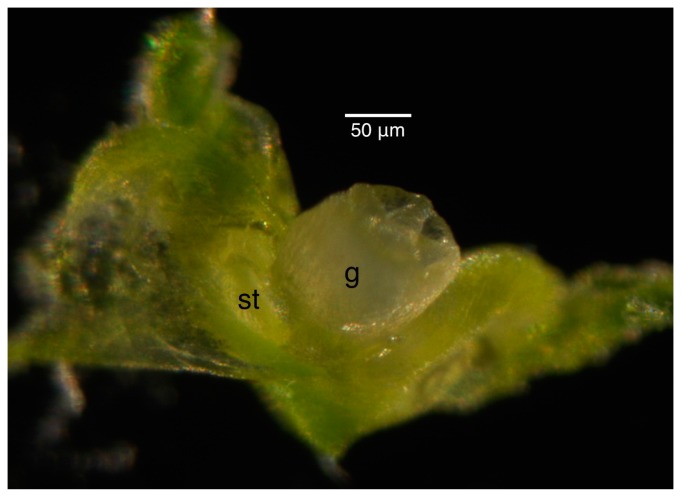
Stage 7 female uninfected floral bud. The gynoecium has a “barrel and star” appearance from the top. Stamen primordia can be observed. Size bar, approximately 50 µm. g, gynoecium; st, stamen.

**Figure 8:**
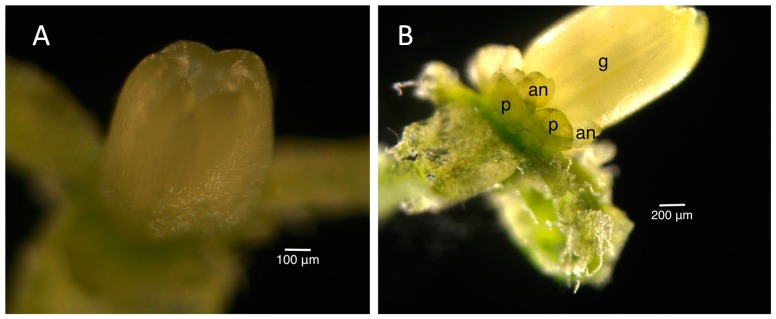
Stage 8 (**A**) and 9 (**B**) of female infected floral buds. Stage 8 had an elongated “barrel and star” appearance, while the gynoecium of the Stage 9 bud was closing up on top. In addition, the bilobal anthers were visible, and the petals were tongue-like shaped in the Stage 9 bud. Size bars, approximately 100 and 200 µm, respectively, in (**A**,**B**). g, gynoecium; p, petal; an, anther.

**Figure 9:**
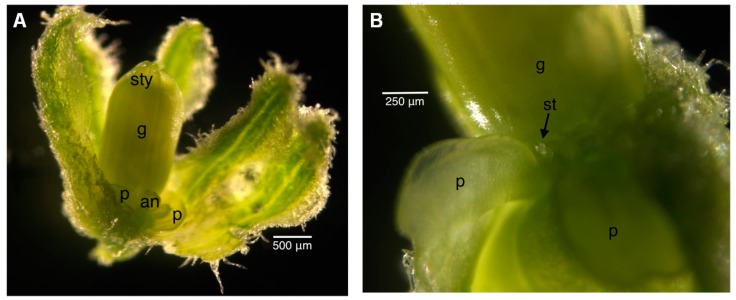
Stage 10 of female infected (**A**) and uninfected (**B**) floral buds where the styles start to appear. In the infected bud, the anthers continue to develop, while the stamen in the uninfected bud becomes rudimentary. Size bars, approximately 500 and 250 µm, respectively, in (**A**,**B**). sty, styles; g, gynoecium; p, petal; an, anther; st, stamen.

Table 1 and Table 2 provide an inventory of staging for infected and uninfected female flowers, respectively. In the female plants, there was great variation in anther sizes in the infected plants, thus precluding the type of scatterplot analysis used for the male flowers. In the reference literature [9,10,27], the appearance of the gynoecium was consistent at each stage regardless of infection status; hence, this was used as the point of reference for staging. The primordia of the gynoecium appeared to be “mushroom”-shaped at Stage 6, as in the male bud (Figure 2). The “barrel and star”-shaped gynoecium can be observed in Figure 7 at Stage 7. The “barrel and star” then elongated (Figure 8A) at Stage 8 before it started to close up (Figure 8B) at Stage 9. Styles then started to appear (Figure 9A) at Stage 10. Although we attempted to observe the size of the anthers and petals, there was no correlation to the gynoecium stage.

**Table 1:** Size observation and staging of female infected floral buds from two plants.

Bud Size (Rough)	Bud Size (Scope)	Shape of Gynoecium ^a^	External Anthers	Internal Anthers	Petals	Stage
<1 (in sepal of 1.5 mm)	-	Petal and stamen primordia start to develop				5
<1 (in sepal of 2 mm)	-	“mushroom” shaped with petal primordia separated from internal stamen primordia	-	-	Not visible	6
<1	0.8	-	Unilobal	-	Not visible	7
<1	0.7	Shallow “barrel and star”	0.1 mm bilobal	0.05 mm unilobal	Not visible	7
0.5	0.7	“barrel and star” shape	0.075 mm bilobal	0.05 mm unilobal	Not visible	7
1	1.4	elongated “barrel and star”	0.25 mm tetralobal	0.15 mm bilobal	Not observed	8
1	1.5	elongated “barrel and star”	0.1 mm bilobal	Not observed	Not visible	8
1.5	1.75	elongated “barrel and star”	0.45 mm tetralobal	0.35 mm tetralobal	0.2–0.3 mm cordiform	8
2	1.75	gynoecium beginning to close	0.35 mm tetralobal	0.25 mm tetralobal	small tongue-like	9
2	2.25	gynoecium beginning to close	0.2 mm tetralobal	Not observed	tongue-like	9
3	2.5	gynoecium beginning to close	0.45 mm tetralobal	0.3 mm tetralobal	0.4 mm tongue-like	9
3	2.5	Styles appearing	0.2 mm tetralobal	Not observed	Overlapping	10
3.5	3.25	elongated gynoecium and closing of “barrel”	0.65 mm tetralobal	0.65 mm tetralobal	1 mm slight cordiform	9–10
3.5	3.25	Styles appearing	0.65 mm tetralobal	0.55 mm tetralobal	0.7 mm cordiform	10
3	3	Styles appearing	0.7 mm tetralobal	0.6 mm tetralobal	0.7 mm cordiform	10
4	3.5	Style appearing	0.2 mm tetralobal	Not observed	0.75 mm	10
4	4	Visible styles	0.75 mm tetralobal	0.6 mm tetralobal	1 mm cordiform	10
5	5	Elongation of styles	0.35 mm tetralobal	Not observed	1.25 mm	>11
5	5.5	Short style and stunted gynoecium	1.2 mm tetralobal	0.8 mm tetralobal	2.5 mm full petal	-
7	7.5	Elongated gynoecium with stunted style	2 mm tetralobal	1.25 mm tetralobal	5.5 mm full petal	>11
8	8.5	Long style	0.75 mm tetralobal	0.25 mm tetralobal	4 mm full petal	>11

^a^ Figure 2 illustrates the “mushroom”-shaped gynoecium. Figure 7 illustrates the “barrel and star”-shaped gynoecium. Figure 8 illustrates the elongation and closing of the “barrel and star” gynoecium. Figure 9A shows the styles appearing. Figure 4A illustrates the tongue-like petals, and Figure 4B shows the cordiform petals.

**Table 2:** Size observation and staging of female uninfected floral buds from four plants.

Bud Size (Rough)	Bud Size (Scope)	Shape of Gynoecium ^a^	Petals	Stage
<1	0.7	“barrel and star” shape	Visible and not overlapping	7
<1	0.7	“barrel and star” shape	Hardly visible	7
1	1.1	“barrel and star” shape	Visible and larger than others	7
1	1.2	elongated “barrel and star”	Tongue-like	8
Slightly > 1 mm	1.5	elongated “barrel and star”	0.7 mm	8
1	1.5	elongated “barrel and star”	Cordiform	8
1.5	1.5	elongated “barrel and star”	Hardly distinguishable from anthers	8
2	1.75	elongated “barrel and star”	Visible	8
2	2	elongated “barrel and star”	Visible and not overlapping	8
2	2	elongated “barrel and star”	No petal	8
2.5	2.5	elongated “barrel and star”	0.2 mm transparent cordiform	8
2.5	2.75	gynoecium beginning to close	0.3 mm cordiform	9
3	2.5	Styles appearing	Visible and not overlapping	10
3	3	Visible styles	0.35 mm cordiform	10.1
4	4	Styles appearing	Visible and overlapping	10
4	4	Visible styles	0.75 mm cordiform	Mid 10
4	4	Visible styles	0.75 mm cordiform	10.2
4	4.5	Visible styles	0.9 mm cordiform	10
4.5	4.5	Visible styles	1.5 mm fully formed	10
5	5	Long styles, petal half of ovary	1.5 mm half of ovary	10
6	6	Long styles, petal as long as ovary	2.5 mm, covers ovary	>11
7	7	Long styles, petal as long as ovary	3.5 mm, covers ovary	>11
7	6	Long styles, petal as long as ovary	1.75 mm fully formed	>11

^a^ Figure 2 illustrates the “mushroom”-shaped gynoecium. Figure 7 illustrates the “barrel and star”-shaped gynoecium. Figure 8 illustrates the elongation and closing of the “barrel and star” gynoecium. Figure 9A shows the styles appearing. Figure 4A illustrates the tongue-like petals, and Figure 4B shows the cordiform petals.

In the female plants, the divergence from published literature [27] was a little more pronounced, although the floral bud size for each stage was consistent between the infected and uninfected plants (Table 1 and Table 2). For practicality in tissue collection, we collected the buds smaller than 1 mm, often found in the sepal, as one category. In the reference literature [27], female buds of this size should be at Stage 6 and below. In our buds, however, they had already reached Stage 7 (Figure 7).

Buds ranging from 1.0–2.9 mm appeared to be in Stages 8 (Figure 8A) and 9 (Figure 8B) of floral development. Again for practical reasons, it was not possible to separate the buds in Stage 8 from Stage 9 for tissue collection purposes. In the reference literature [27], these buds should only be at Stage 7. Although the petal size should be above 1 mm by Stage 8, the petals in our buds were much smaller. We would attribute this to the fact that the overall size of the bud was much smaller than in the reference literature [27]; hence, a larger petal would not be possible.

Buds ranging from 3.0–4.9 mm appeared to be in Stage 10 (Figure 9) of floral development. This is much smaller than expected compared to the reference literature [27], where Stage 10 buds range from 8–11 mm in size. Floral buds 5 mm and beyond were categorized as late stage.

To confirm that our size range was valid, we looked into previous studies of this pathosystem that involved smutted floral buds. A comparison of healthy and infected male and female gene expression used floral buds of a size of 1–3.5 mm for both infected and uninfected buds and determined these to be Stages 8–11 [24]. In another study to identify MADS box genes in the early stages of floral development up to Stage 10, floral buds of less than 4 mm were used in the generation of their cDNA library [23]. These previous studies gave us confidence that we had staged the floral buds accurately.

### 2.3. RNA Extraction

Collected floral bud tissues were processed using a previously-published protocol [25]. Table 3 is a summary of the estimated weight of the collected tissue and amount of crude RNA extracted. The amount of RNA yield was dependent on the amount of tissue used, as well as the number of extraction columns used. While each extraction column can extract RNA from up to 100 mg of tissue, not every sample had that much tissue for extraction. Moreover, since only about 40 μg of RNA were needed for all quality assessment and RNA-sequencing, not all ground tissue was processed.

Although all of the tissue submitted for RNA-sequencing met the integrity requirements of the Broad Institute where RNA-Seq was performed, not all of the samples produced a good RNA integrity number (RIN). We hypothesize that the RIN tool may not have been optimized for the electropherogram of plant and fungal samples, since it was trained with human, mouse and rat RNA [39]. It should also be noted that the integrity of the RNA does not necessarily reflect the accuracy of staging.

### 2.4. Unusual Floral Buds

**Figure 10:**
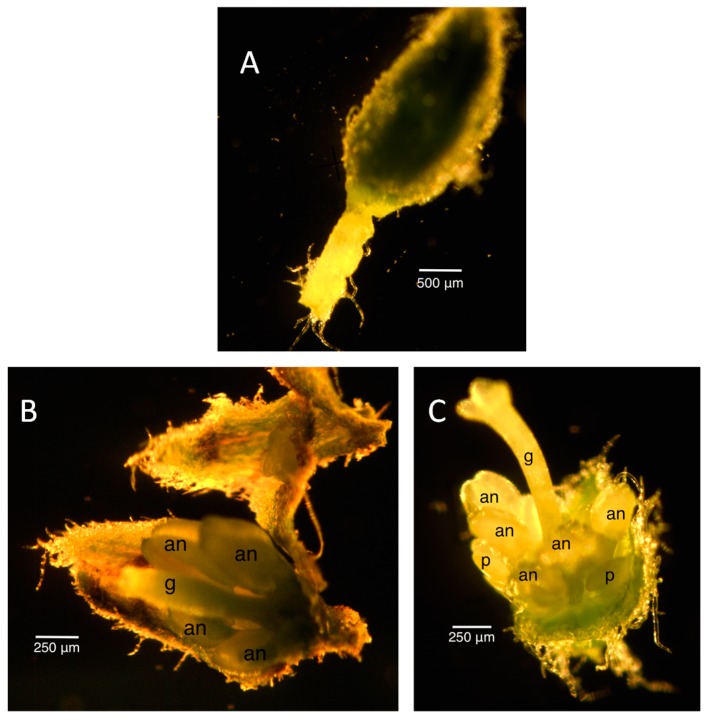
Unusual floral bud morphologies. (**A**) Segmented pedicel with buds that die prematurely; (**B**,**C**) Male floral buds that develop a pseudo-gynoecium. Size bars, approximately 500 µm in (**A**) and 250 µm in (**B**,**C**). an, anther; g, gynoecium; p, petal.

There were many difficulties achieving stable infection where plants consistently produced smutted flowers. In the course of monitoring the plants, we noticed some morphologies that differed from the “normal” infection phenotype. Some of these included plants that remained in the rosette stage or floral buds that appeared, but never bloomed (Figure 10A). We do not know whether these variations in morphology were sex-dependent, but the infected female plants tend to develop more variable phenotypes, such as the early death of buds. There were also some gross distortions in flowers that made it impossible to tell the gender unless a genetic analysis was performed or a “normal” flower presented itself on the plant at some point. Some examples of these distortions include: exposed smutted anthers with no calyx and petals (assumed male); stunted ovaries with no obvious smutted anthers (assumed female); infected male flowers developing pseudo-gynoecium (Figure 10B,C); a robust ovary that can set seeds with smutted anthers (assumed female).

**Table 3:** Summary of RNA extracted from floral bud tissue based on the stages of interest.

Tissue Type	Stage	Weight of Tissue (mg)	Amount of Crude RNA (μg)	RNA Integrity Number (RIN) ^a^
Male infected	8	91	64	N/A
Male infected	8	38.2	62	9.4
Male infected	9	150.6	61	N/A
Male infected	9	101.5	85	8.9
Male infected	10	116.2	180	9
Male infected	10	110.2	115	9
Female infected	7	34.7	51	6.4
Female infected	9	55.2	91	6.4
Female infected	10	187.8	143	9.3
Male uninfected	8	28	45	6.9
Male uninfected	9	34.4	40	6.1
Male uninfected	9	19.2	63	N/A
Female uninfected	9	30	104	6.3
Female uninfected	10	90	103	6.2
Female uninfected	10	109	120	N/A

^a^ RIN as determined for the Eukaryotic RNA chip for BioAnalyzer, using mammalian systems as the standard for rRNA positions and integrity.

At this point, we are uncertain if these unusual phenotypes were due to mutation(s) in the pathogen as in the petalless flower [30] or if they reflect a physiological defensive mechanism of the host [33], as a trade-off of being diseased [34]. Alternatively, perhaps they reflect the incompatibility of the host and the pathogen [37]. There has not been a systematic inventory published of all possible phenotypes of disease in this pathosystem, especially where each “phenotype” was only found in one plant. It would be interesting to investigate the genetic/gene expression basis of the variation as, for example, in the petalless flower [30].

## 3. Experimental Section

### 3.1. Germination

*Silene latifolia* seeds were harvested in summer 2009 from greenhouse-grown plants; these were originally from seeds of a field population in Clover Hollow (37.328–80.488) near Mt Lake Biological Station, Virginia, and were kindly provided by Michael E. Hood. Seeds were sterilized and hydrated by soaking in a sterilizing solution (40% household bleach, 20% absolute ethanol and 1 drop of triton X-100 as surfactant per 50 mL of solution) and washing five times in sterile distilled water, for 2 min per wash with constant agitation. Each seed was then individually planted in closed milk jars on sterile 0.3% phytagar (Thermo Fisher, Pittsburgh, PA, USA), 0.5× MS (Murashige and Skoog) salt (Sigma-Aldrich, St. Louis, MO, USA) and 0.05% MES (2-(*N*-morpholino)ethanesulfonic acid) buffer (Thermo Fisher). Each jar was incubated at 4 °C for 5 days to synchronize germination before transferring them to the growth chambers. In accordance with the growth condition and its effect (unpublished data) on the maintenance of flowering status and infection in its hosts, we maintained the growth chamber at 20 °C with 13 h of daylight. Germination started within 3 days with the appearance of the radicle. When the seedlings were about 15 days old, they were transferred into 2 inch square pots filled with Sunshine MVP Professional Growing Mix (Sun Gro Horticulture Canada Ltd., Agawam, MA, USA, Cat No. 02392868) and replaced into the growth chamber. Humidity was kept high initially using dome covers and flood trays. Seedlings were gradually exposed to the chamber environment for increasing amounts of time daily in order for the seedlings to harden and adapt to the lower humidity. The plants were repotted to 4 inch diameter round pots when they began to bolt, at about 30 days old. They were further repotted into 7 inch diameter round pots when they had almost attained maximum height or when the volume of soil was not sufficient to provide hydration for the plant. The plants were watered every other day with 100-ppm fertilizer (Peters Professional 15-16-17 Peat-Lite Special^®^ Formula, No. S12893, Dublin, OH, USA) to wet the soil thoroughly.

### 3.2. Infection with Microbotryum lychnidis-dioicae

Haploid fungal cells (p1A1 and p1A2) (“Lamole strain”: GenBank 100-15Lamole; [25,37,40]) grown under “rich” conditions were allowed to grow for 4–5 days on yeast peptone dextrose media (YPD; 1% yeast extract, 10% dextrose, 2% peptone, 2% agar) at room temperature. To prepare pre-mated mixtures, the cells were harvested and resuspended in sterile distilled water, adjusted to a concentration of 1 × 10^9^ cells/mL in equal proportion before being spotted onto nutrient-free solid agar media (2% agar) in 50-μL spots. The plates were allowed to dry and then incubated at 14 °C for about 48 h. Cells were inspected for conjugation tubes under the microscope before being harvested for infection purposes. To prepare unmated mixtures, the cells were harvested and resuspended in sterile distilled water in equal proportion before being used directly for infection purposes. For infection, various permutations of treatments were explored, including pre-mated *vs.* unmated cells, infection on different days after germination (3, 10, 12, 21, 28 days after germination and dual-infection for some), injection *vs.* inoculation (placing the cell suspension on the central whorl) of meristem and various volumes and concentrations of fungal cells used. For staging purposes, the method of infection was not taken into account. Instead, robustly-infected and uninfected plants that could be used for tissue collection were staged specifically for RNA-Seq purposes. The gender, infection status and the growth chamber where the plant was located were noted as possible factors that might affect the size of floral buds and their developmental stage. Infection status was determined by the consistent blooming of fully-smutted flowers, and for uninfected plants, we used the control plants that were not in any way inoculated with the fungus and that grew in a separate chamber from infected plants.

### 3.3. Floral Bud Collection and Examination

At the time of collection, each inflorescence was separated using forceps and hand-categorized at 1-mm intervals based on a marked scale on the collection plate. Each collection was limited to 30 min on a single plant, to minimize dehydration of the bud before measurement under the dissecting scope. All floral buds were examined as fresh tissue.

The floral buds were then staged according to previous literature [9,27] under the dissecting scope (Nikon, Melville, NY, USA, Model: SMZ-U). For male buds, both infected and uninfected, the stages were determined by the petal and size of the external and internal anthers. For female plants, the shape of the gynoecium was the major determinant. These measurements were made with a glass stage micrometer (Imaging Research, Inc., St. Catherines, ON, Canada) under the dissecting scope. The results were tabulated to determine if there was a correlation of floral bud size to its developmental stages across different plants.

## 4. Conclusions

In this study, we were able to correlate stages of flower development to bud size in both uninfected and infected male and female *S. latifolia* plants, a critical aspect of conducting reproducible analyses of differential transcriptome studies for discrete stages of late infection by *M. lychnidis-dioicae*. This was critical to our later large study of differential gene expression during these stages of development, for both the host plant and the fungus [26]. As an example, comparison of Stage 8 with Stage 9 for infected male flowers indicated differences for approximately 140 *M. lychnidis-dioicae* genes, with a false discovery rate (FDR) <10^−5^. One of the more commonly observed changes was upregulation of genes in the MFS (major facilitator superfamily) family of transporters. In addition, in these preliminary analyses, we observed upregulation of some members of the glycosyl hydrolase family and other pectinesterases associated with host plant cell wall degradation. Such preliminary results support the efficacy of the type of staging we used in the current study.

Throughout this study, we encountered and overcame difficulties obtaining reliable systemic infection, another necessary aspect of the RNA-Seq work comparing infected to uninfected plants. As part of this study, a subset of host plants was not used for collection purposes for sizing and RNA-Seq, as they exhibited some symptoms that did not fit the classic characteristics reported previously for infections in the *S. latifolia/M. lychnidis-dioicae* system. We hypothesize that the unusual plant symptoms we observed may be part of the plant’s physiological defense strategy against the fungus [33] or possible incompatibility between the host and pathogen [37]. By suppressing bolting (remaining as a rosette), flowering (non-flowering bolts) or blooming (pre-mature death of buds), the host would present an environment where the fungus would be unable to propagate and complete its sexual life cycle through teliosporogenesis in the anthers. Another plausible hypothesis is that a non-optimal infection method resulted in a hyper-infection that interfered with the normal plant development. Although this would ultimately prevent the fungus from completing its lifecycle, such cases of apparently counter-productive hyper-virulence have been observed in other systems [41,42]. One possible way to address aspects of these unusual plant symptoms in the future will be to conduct RNA-Seq on tissues from such plants to examine changes in the transcription levels of potential target genes associated with development (e.g., MADS-box genes).

The simple approach in staging the floral buds developed in this study allowed us to quickly determine the correct floral bud sizes to collect. While the male buds were not too different from the documented literature, the female buds differed considerably. Failure to perform the in-house staging would have resulted in missing the early stages of fungal development in the floral buds used for the extensive transcriptome analyses.
